# Supplementary material for: Auditory detection is modulated by theta phase of silent lip movements
Source: Curr Res Neurobiol. 2021 Jun 12;2:100014. doi: 10.1016/j.crneur.2021.100014 (PMC9559921; doi:10.1016/j.crneur.2021.100014)
Supplement: Multimedia component 4 [file mmc4.docx]

**Tone detection performance and visual entrainment in the single tone condition.**


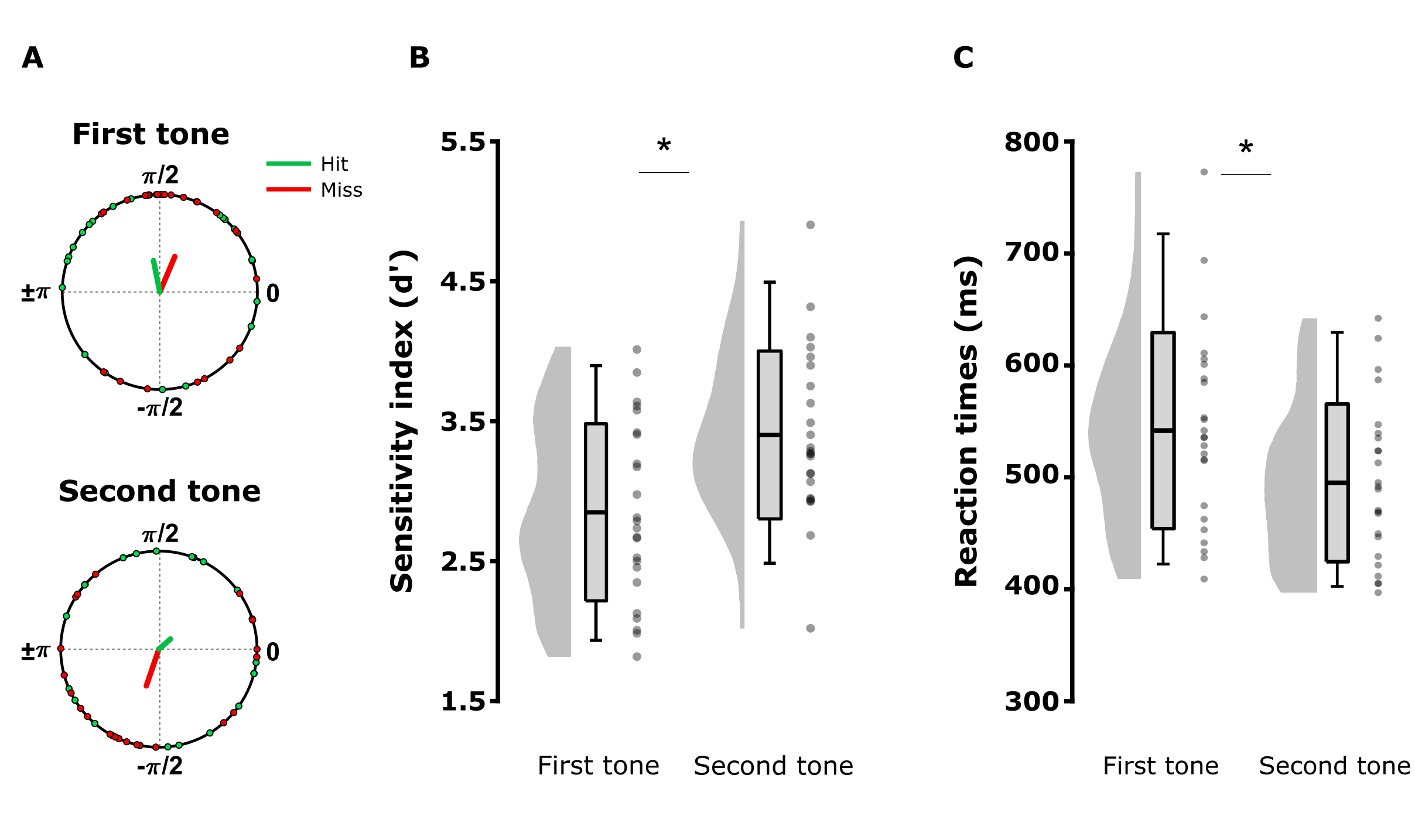


**Figure S1: Visual entrainment and tone detection performance in the single tone condition.**

Although in this condition there was only one tone per trial, we sorted each single tone as a first or a second tone according to its onset. (A) Resultant vector length r (green line) from grand average phase at the onset of first tones (green line: hits; red line: miss) and second tones (green line: hits; red line: miss). The individual mean theta phases are depicted in polar coordinates (green and red circles, respectively for hit and miss trials). (B) Mean sensitivity index (d’) and (C) reaction times. The graphs depict the density, the grand average (mean ± standard deviation; errors bars indicate 5^th^ and 95^th^ percentiles), and individual means (grey dots) for tones sorted as first or second ones. Significant contrasts are evidenced with stars.

To control that the better detection of the second tone did not simply reflect an attentional effect driven by the occurrence of the first tone in the two tones condition (Figure 3B), we compared performance with the single tone condition by sorting each unique tone of the trial as a first or second tone according to its onset (i.e. respectively between 0 and 2.5 s or 2.5 and 5s after trial onset; Figure S1 B&C). Two independent one-sample t-tests established that participants detected the first and second tones in the single tone condition, with d’ scores greater than zero (first tone: T(1,23) = 21.99; p < 0.001, two-tailed; second tone: T(1,23) = 27.69; p < 0.001, two-tailed). Two paired-samples t-test performed on d’ scores and reaction times confirmed that the second tones were better detected (figure S1 B; T(1, 23) = 5.385; p < 0.001; two-tailed), and faster as compared to the first tones (figure S1 C; T(1, 23) = 4.778; p < 0.001; two-tailed). Further, we compared directly the tone detection performances between the single tone and two tones conditions by mean of 2-by-2 repeated-measures ANOVAs (factors condition and tone position). A main effect of position showed that the second tones were better detected than the first tones in both conditions (F(1, 23) = 37.123; p < 0.001). No main effect of condition (F(1, 23) = 2.302; p = 0.143) or interaction between condition and tone position on d’ were found (F(1, 23) = 0.658; p = 0.426). A repeated-measures ANOVA on reaction times showed a significant effect of tone position with faster responses to second tones than first tones (F(1, 23) = 33.797; p < 0.001). A significant effect of condition showed overall faster reaction times in the two tones condition as compared to the single tone condition (F(1, 23) = 14.047; p = 0.001), but no interaction between tone position and condition (F(1, 23) = 0.173; p = 0.682).

Finally, although initially designed to control for attentional bias on detection performance (and counterbalance the number of trials followed by a second tone to control for its predictability), we tested whether the phase modulation across participants reported at the second tone in the two tones condition would be true also at the second tones in the single tone condition as well (Figure S1 A). Similarly to Figure 3A (see second approach in the Result section), we compared the resultant vector length r (green line) from grand average phase at the onsets of tones sorted as first tones hits (n = 24; µ = 1.769 rad or 101.336°; r_first tone_ = 0.329; p = 0.074) and second tones hits (n = 24; µ = 0.719 rad or 41.165°; r_second tone_ = 0.157; p = 0.558) in the single tone condition. A permutation test on the resultant vector length difference between the tones sorted as first and second tones did not reveal significant difference (Figure S1 A; permutations: 10000; effect size = - 0.171; p = 0.835). Further, the permutation test applied to assess the difference of effect size between the first and second tones ([z-value_hit-miss_]_second tone_ - [z-value_hit-miss_]_first tone_) did not reveal any significant interaction between visual phase modulation and tone detection (permutations: 10000; effect size = -0.172; p = 0.866). Two additional permutation tests on the difference between hits and miss trials (z-value_hit-miss_) revealed an effect of phasic modulation on performance neither in the first tone window (permutations: 10,000; effect size = -0.066; p = 0.633) or in the second tone window (permutations: 10,000; effect size = -0.238; p = 0.985).


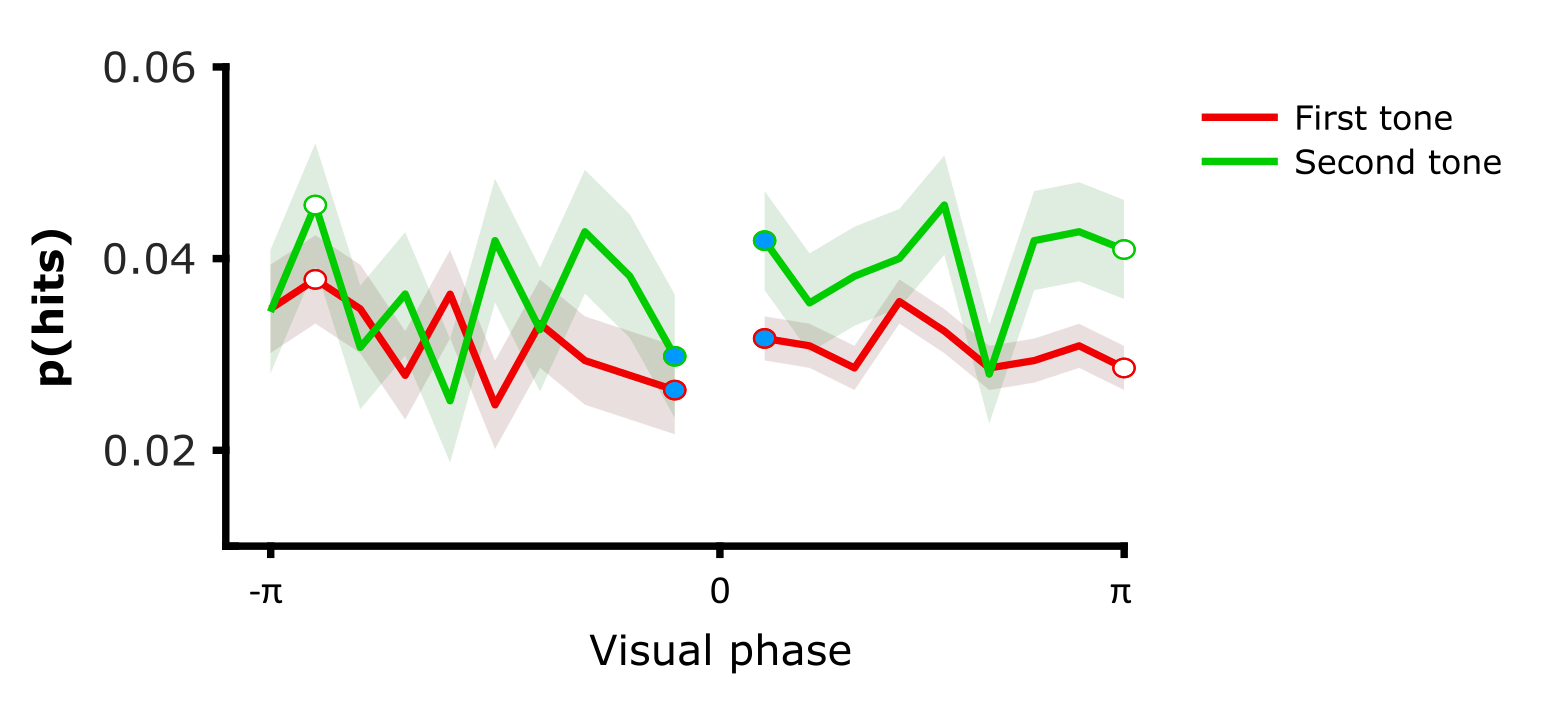


**Figure S2. Phasic modulation along the realigned phase in the single tone condition.** Probability of correctly detected tones p(Hits) = hits / (hits + misses) along the visual phase realigned on the preferred bin (0° bin, not plotted) in the single tone condition. As previously, each single tone was sorted as a first or a second tone according to its onset. The red line depicts p(hits) at the first tone and the green line depicts p(hits) at the second tone (mean ± standard deviation). The phase modulation was estimated by subtracting the average response of the two bins adjacent to the bin opposite to the preferred phase (white dots) from the average response of the two bins adjacent to the preferred phase (blue dots).

Finally, we tested for a phase locking within participants after realigning individual phases on their preferred phase. First, we computed the probability of correctly detecting the tone p(Hits) = hits / (hits + misses) as a single measure of performance, for tones sorted as first and seconds in the single tone condition (Figure S2). We assessed the existence of a phase modulation at detected sorted first and second tones (i.e. hit trials) by testing the mean distance against zero for hit trials at each tone (first and second tones) with one-sample T-tests as previously for the two tones condition (mean phase modulation against zero; one-tailed; p-values adjusted with a Bonferroni correction for multiple comparisons). Results revealed that the mean distance was not significantly greater than zero at the first tones (t(1,23) = -1.035; p*_adjusted_* = 1; Cohen’s d = -0.211) and the second tones (t(1,23) = -1.444; p*_adjusted_* = 1; ; Cohen’s d = -0.295). This result suggested the absence of phase modulation driving the correct detection of the first and second tones in the single tone condition (Figure S2). Second, we compared the amplitude of the phasic modulation between hit and miss trials (i.e. t-value_hit-miss_) at the sorted first and second tones of the single tone condition. We applied the same permutation-based analyses as the two tones condition described in the Result section. The two permutation tests testing the original t-value against the t-values of the permuted data revealed no phasic modulation in the first tone window (permutations: 10,000; effect size = -0.068; p = 0.511), nor in the first tone window (permutations: 10,000; effect size = -0.926; p = 0.995). A third permutation test addressed the interaction between phase modulation and tone position by assessing the statistical difference of phase modulation between the second and first sorted tones, i.e. [t-value_hit-miss_]_second tone_ - [t-value_hit-miss_]_first tone_. Results revealed that the phase modulation was not significantly different between the first and second sorted tones (permutations: 10,000; effect size = -0.495; p = 0.952). Nevertheless, the absence of phase modulation here (both with or without realigning the phase on the individual preferred bin) may be explained by the fact that single tones were sorted as first or second tones according to their onset, leaving only half as many trials as in the two tones condition of interest.

**Distinct preferred phases showed performance differences between two subpopulations of listeners.**

The behavioural data of the TDT task suggests that two separate subpopulations entrained to different preferred theta phases in the second tone time-window (see Figure 3A lower panel and Figure S3B below). In a post-hoc analysis, we assessed whether these apparently distinct populations also showed differences in tone detection performances. Arguably, any difference should be most pronounced only when visual entrainment eventually took place (second tone window) but not early in the trial. Participants were sorted in two groups based on their mean theta phase in the second tone window (i.e. n_group1_ = 11 and n_group2_ = 10; see Material and Methods) and we compared detection performance (d’) in the two tones condition by means of a repeated-measures ANOVA (with factors tone position and group).


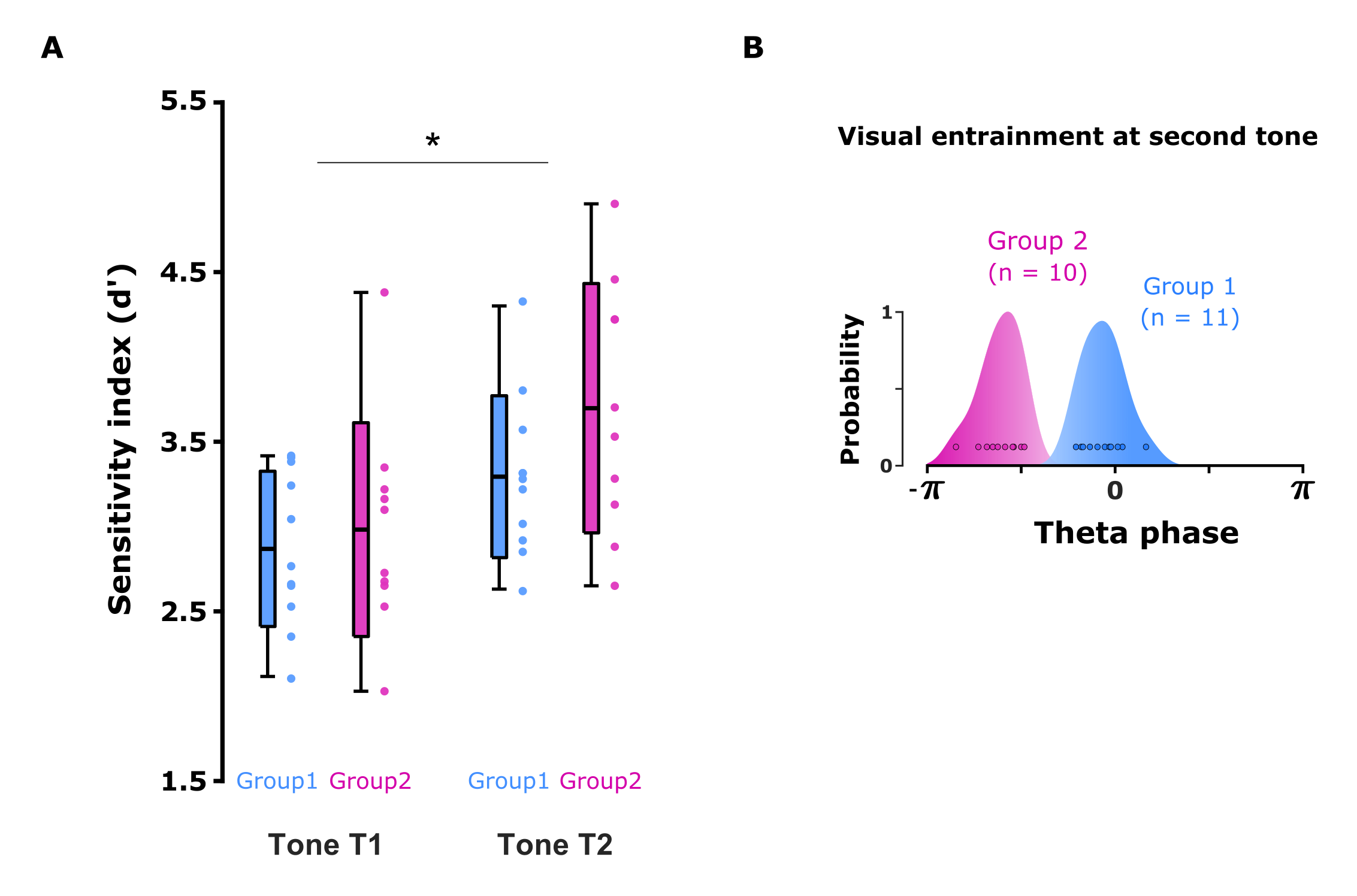


**Figure S3: Preferred theta phase affects tone detection task performance.** (A) Tone detection sensitivity (d’) of first and second tones between the group 1 and 2. The graphs depict the density, the grand average (mean ± standard deviation; errors bars indicate 5^th^ and 95^th^ percentiles), and individual means for the first/second tones. Post-hoc analyses revealed that the effect of tone position on the detection performances (first tone versus second tone) was greater in the group 2 than group 1, suggesting that visual entrainment affected auditory processing differently between the two groups. Significant contrasts are evidenced with stars (p < 0.05). (B) Mean phase distributions of the group 1 (blue) and group 2 (pink). The two separate populations were sorted based on their individual preferred phase at the second tones (blue and pink dots), where visual entrainment supposedly took place.

The ANOVA on *d’* scores revealed a significant interaction between tone position and group (F(1, 9) = 9.224; p = 0.014) revealing that the visual phase modulation affected differently detection performance between the two groups. Bonferroni-corrected pairwise t-tests showed that this interaction was driven by a greater effect size of tone position in the group 2 than in the group 1, although both groups were better at detecting second tones as compared to first tones. Results also replicated the effect of tone position (F(1, 9) = 12.613; p = 0.006) with greater d’ for the tones sorted as second than first. Finally, no main effect of group was found (F(1, 9) = 0.918; p = 0.363). No difference between threshold (SNR_group1_ = 1.39e10^-3^ ± 2.31e10^-3^; SNR_group2_ = 1.43e10^-3^ ± 3.47e10^-3^; T(1, 24) = - 0.66; p = 0.95; two-tailed), nor hit rates (hit_group1_ = 0.761 ± 0.003; hit_group2_ = 0.763 ± 0.004; T(1, 24) = -0.23; p = 0.84; two-tailed) were found in the calibration task, ruling out any hearing difference.

**Stimuli analyses**


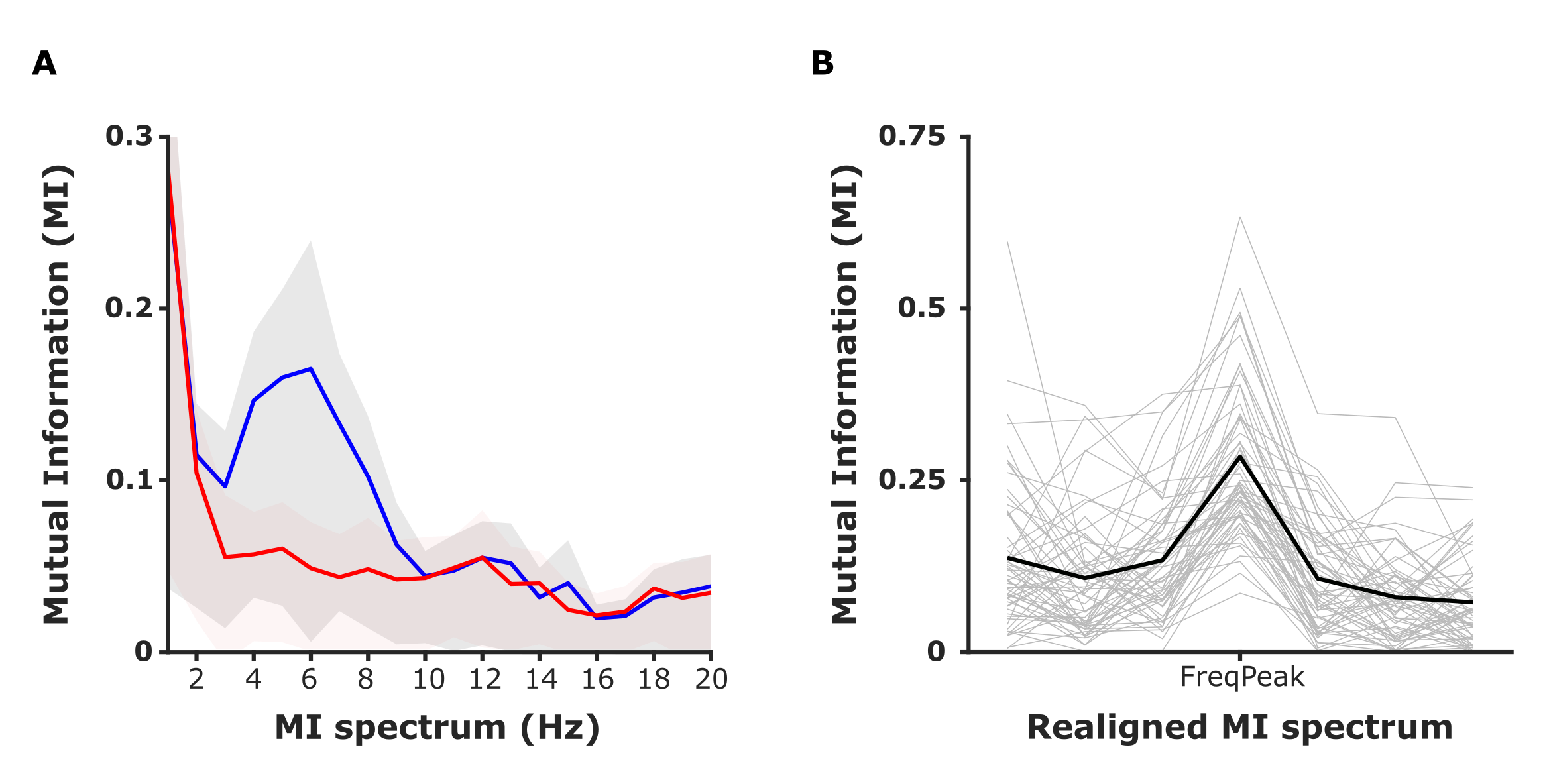


**Figure S4: Mutual information between lips movements and auditory envelope in the movies.** (A) Mean mutual information spectrum (± standard deviation) between the vertical aperture of the lips and the corresponding (blue line) or random (red line) speech envelope from movies. The greater dependency between the two signals is reflected by the bump localised in the theta frequency band of interest (4 - 8 Hz). (B) Realigned spectrum on the frequency with the greater MI peak (± 1-3 Hz) of each movie (grey lines) and averaged (black line). For each movie, we applied a peak detection and selected the stimuli with a greater MI between the vertical aperture of the lips and auditory envelope situated in the frequencies of interest only (i.e. 4, 5, 6, 7 and 8 Hz).
