# Supplementary material for: Auditory detection is modulated by theta phase of silent lip movements
Source: Curr Res Neurobiol. 2021 Jun 12;2:100014. doi: 10.1016/j.crneur.2021.100014 (PMC9559921; doi:10.1016/j.crneur.2021.100014)
Supplement: Multimedia component 1 [file mmc1.pdf]

Peer Review Overview

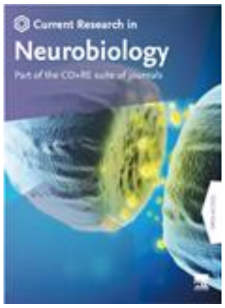

Manuscript Title: Auditory detection is modulated by theta phase of silent lip movements

Received	Nov 21, 2020
1st Decision	Feb 08, 2021
1st Revision Submitted	Mar 08, 2021
2nd Decision	Mar 31, 2021
2nd Revision Submitted	Apr 16, 2021
3rd Decision	May 06, 2021
3rd Revision Submitted	May 12, 2021
Accepted	May 19, 2021

1st Decision letter

Reference: CRNEUR-D-20-00016
Title: Auditory detection is modulated by theta phase of silent lip movements
Journal: Current Research in Neurobiology

Dear Dr. Biau,

Thank you for submitting your manuscript to Current Research in Neurobiology.

I have completed my evaluation of your manuscript. The reviewers recommend reconsideration of your manuscript following major revision. I invite you to resubmit your manuscript after addressing the comments below. Please resubmit your revised manuscript by Apr 09, 2021.

When revising your manuscript, please consider all issues mentioned in the reviewers' comments carefully: please outline every change made in response to their comments and provide suitable rebuttals for any comments not addressed. Please note that your revised submission may need to be re-reviewed.

Current Research in Neurobiology values your contribution and I look forward to receiving your revised manuscript.

Kind regards,

Anna S Mitchell, Ph.D.
Editor in Chief
Current Research in Neurobiology

Comments from Editors and Reviewers:

Thank you for submitting your manuscript. Two experts in the field have reviewed your manuscript. Please see their suggestions and queries outlined below and address them in the revision of your manuscript. Please also consider providing a graphical abstract to visually illustrate the main message in this manuscript.

Reviewer #1:

I have previously reviewed this manuscript for another journal. Some of the text below is still relevant and has therefore been reproduced from my initial review.

The present manuscript examines the influence of visual information on auditory perception during human speech processing. In particular, the authors test whether the quasi-rhythmic lip movements during silent speech movies modulate the detection of simple auditory targets (one or two pure tones). Indeed, they report that auditory perception depends on the timing - i.e. phase - of the visual input. A separate EEG experiment suggests that visual information drives auditory processing, indicated by stimulus-related neural activity in visual regions leading that in auditory ones.
The approach taken by the authors is highly interesting and timely, and could be a major influence in the corresponding research field. However, several issues with the paper, raised in my previous review, persist. These are described below.

1) The authors describe their findings as evidence for theta oscillations "organising information flow across visual and auditory sensory areas". I wonder whether the results reported can simply be explained by the fact that visual and auditory systems are connected with each other, i.e. visual input will also be transmitted to auditory regions, and vice versa. If the authors provide quasi-rhythmic input to the visual system (in the form of lip movements), then this information will also be relayed to the auditory system - potentially with a certain time lag (reaching the visual system first, given that the stimulus is visual). If the two systems are connected, then it is likely that input from one modality will influence the processing of simultaneous information in the other. It might therefore not be very surprising that the detection of auditory targets depends on the timing of simultaneous visual input: For instance, auditory detection might be worse when visual input is particularly informative. If the latter changes rhythmically, then its influence on the former will also be rhythmic.

The authors state that "[their] results show that the theta phase lag between auditory and visual cortices decreases across time, which speaks against a process that would just passively relay activity from visual to the auditory cortex". However, the first (but not the second) time window will be influenced by neural responses to stimulus onset. Steady-state responses to rhythmic stimuli typically take some time until they follow the input rhythm in a more stable fashion. The fact that the phase lag between auditory and visual regions changes over time might simply reflect the neural response becoming more stable after an initial period influenced by stimulus onset, and does not necessarily provide evidence for an active mechanism preparing the auditory system for upcoming information. Indeed, the fact that the phase of lip movements modulates auditory detection even in a scenario in which visual and auditory information are unrelated, seems to speak against such a mechanism, and rather for a more automatic one.

In addition, it is well known that auditory perception is optimal when preceded by visual input within a certain time window (30-100 ms) - see for instance work by Thorne & Debener, Diederich & Colonius. It seems to me that the authors replicate this work, with the only difference being that their visual information fluctuates quasi-rhythmically - leading to benefits on auditory perception that are also quasi-rhythmically.

Together, I believe that these issues should be given much more prominence in the paper.

2) I was previously confused by the structure of the Results section and the description of statistical analyses, and I still am. It seems to me that the analysis of the main hypothesis - that auditory detection is modulated by the timing (i.e. phase) of lip movements - is hidden in the text. Instead, the authors start by testing whether the distribution of the phase of lip movements during detected auditory targets is non-uniformly distributed across participants. This test does not reveal the magnitude (or indeed, the presence) of a phasic modulation, but instead whether all participants have the same "preferred" visual phase (leading to highest auditory accuracy). The latter could be true even in the absence of a significant phasic modulation. Conversely, even if preferred phases are uniformly distributed, there can still be a phasic effect, but with different "preferred" phases across participants. In my view, the results section needs to start with a statistical quantification of phasic modulation for each of the two tones separately, and in the one tone condition (followed by the comparison across tones). By adapting the analytical approach accordingly, this can be done with or without the assumption that all participants have the same preferred phase. In the Results section, the authors mention this assumption. In the last paragraph of the Discussion, they provide an argument against it: "one could assume that our results may reflect similar anatomical differences that influence how participants synchronize to visual lip activity and how information travels from visual to auditory cortex." Consequently, providing results for both might be the easiest solution. For example, in the authors' "control analysis" (which in my view should rather be a main analysis), they could leave out the alignment step (step two) to include the assumption of identical preferred phases across participants. Alternatively, the authors could fit a sine wave to the data (auditory detection as a function of visual phase) and extract its magnitude as an indicator of phasic modulation, with the phase of the sine wave fixed (assuming identical preferred phases) or variable (assuming different preferred phases) across participants.

From the authors' description, it is currently still unclear to me whether vector length is computed separately for each participant (quantifying phasic modulation of auditory detection for each participant) or for the distribution of mean phases across participants (testing whether these are uniformly distributed). As mentioned above, this makes a big difference for the conclusion that can be drawn from the outcome.

3) A minor point which I would ask the authors to consider: I wonder whether indeed the visual influence on auditory information processing builds up over time during speech processing. Wouldn't that mean that we struggle to understand each beginning of a sentence?

Reviewer #2:

Summary
This paper describes two experiments (a behavioural and an EEG) investigating the effect of silent visual speech on auditory perception. In the behavioural study participants detected tones embedded in noise whilst looking at silent videos of speakers producing speech. Participants were better and faster at detecting tones when they occurred later in the course of the trials. Furthermore, the phase of the

ongoing lip aperture signal at the onsets of successfully detected tones was more consistent later in the course of the trials. This suggested that the quasi-rhythmic lip movements in the silent speech videos may have entrained the visual and eventually the auditory system aligning its excitability with the visual signal. The EEG experiment confirmed this hypothesis demonstrating that the visual speech signal entrained brain activity predominantly in left auditory and visual areas. Phase coupling analysis revealed that the phase of the neural activity in visual areas consistently preceded the phase in auditory areas confirming that visual areas entrained by the silent speech signal modulated activity in auditory areas. These results provide further evidence supporting the hypothesis that visual speech signals improve speech perception via oscillatory mechanisms.

Critique

The objective and main question of the study is timely and interesting. The results represent a relevant contribution to our understanding of the mechanisms of how visual speech signals improve speech perception. I could not identify any serious flaws with the study design and execution. Statistical methods were adequately chosen. The results are adequately discussed in light of a broader body of literature. Figures are well designed, informative and include individual data points. Accordingly, I only have minor comments, mainly aimed at improving clarity, which I list below.

Minor Comments

1. Figure 2:
a. Panel B y-axis tickmarks: 4 units are divided into 3 equal parts between 1.5 and 5.5, this way it is very hard to judge the values. I suggest spacing tickmarks 1 unit apart, so it is easier to judge the values
b. Boxplots throughout the paper: it is not clear if the SEM is depicted by the shaded area around the mean or the box? I suspect the latter, but then what do the shaded grey areas around the mean values represent? Please clarify.
2. I thought that the control experiment described between lines 142 and 160 might be better placed in the supplement, however I do not have strong feelings about this, it is up to the Authors to decide.
3. Line 184-185: It is not clear how this statement follows from the results presented immediately before. Please clarify.
4. Figure 3:
a. Panel A: please indicate the z coordinate of the slice depicted either in the figure or in the legend (I believe it is z = 0)
b. I think it would be informative to indicate the maximal auditory and visual voxels with markers on panel A using the colour coding in panel B.
5. Line 653-655: I did not fully understand why this step was performed the way it is described. Could you please explain in more detail?
6. Line 770: Please indicate which regions were actually included in the auditory and visual areas
7. Line 790: It is confusing which auditory and visual signals are you referring to here. I suspect they are the reconstructed source level time series activities measured at voxels exhibiting maximal MI with the lip aperture signal in auditory and visual areas respectively. Please define this here more precisely.
8. Line 806: The phase slope index calculation is introduced to control for potential circularity issues. Could you please explain briefly here how the phase slope index helps controlling for these issues?

Typos:
1. Line 85-86: "… silent movies centred on the speakers' nose displayed with sound albeit non-informative". I think some words are missing here, please reformulate.
2. Line 88: "mouth apertures" instead of "mouth's apertures"
3. Line 96: "In each trial" instead of "For each trial"

4. Line 97: "for five seconds" instead of "during five seconds"
5. Line 107: "proportion" instead of "percentage"
6. Line 109: "random uniform distribution" instead of just "random distribution".
7. Line 110 and 112: please use either "tone onset" or "tone's onset" consistently
8. Line 182: Please use either Fig. or Figure consistently when referring to figures in the text.
9. Line 196: "… and tone detection improved both in the late window…" It is not clear to me what "both" is referring to here. Please clarify.
10. Line 203: "Sensitive" instead of "sensible"
11. Line 217: "… contrasting the difference of MI between the early and late time window" I may have misunderstood this but to me the sentence would make sense either as: "... contrasting the difference of MI between the early and late time window with zero" or just simply "…contrasting the MI between the early and late time window". The same issue appears in line 744 as well.
12. Line 312: "with" instead of "within"
13. Line 398: a brief reference of where to find the relevant details would be helpful here. Something like: for details see Tone detection performances section.
14. Line 505: "tone detection" instead of "tones detection"
15. Line 509: "consisted of" instead of "consisted in"
16. Line 567: "practice" instead of "practiced"
17. Line 585: "…to reflect the direction of the auditory system…" This sounds a bit odd, please reformulate.
18. Line 638: "… to determine a resultant vector length…" I would perhaps write "…to determine the resultant vector length across participants..." Adding "across participants" would help understanding.
19. Line 664: "where" instead of "were"
20. Line 754: "realigned with respect to" instead of "realigned respect to"

Comments to the Custom Review Question(s) point D:
The data required to replicate the results were not deposited to open data repositories at the time of the review but there is a statement that the data will be made available after publication of the manuscript.

1st Author Response Letter

Response to comments from Editors and Reviewers:

We would like to thank the editor as well as the two reviewers for their constructive comments, which helped to improve the manuscript. Please find our answers to each comment below, where we also explain the corresponding adjustments made in the new version of the manuscript. We hope that we have addressed every concern satisfactorily.
Emmanuel Biau and Simon Hanslmayr, on behalf of all the authors.

Comments from Reviewer 1

I have previously reviewed this manuscript for another journal. Some of the text below is still relevant and has therefore been reproduced from my initial review. The present manuscript examines the influence of visual information on auditory perception during human speech processing. In particular, the authors test whether the quasi-rhythmic lip movements during silent

speech movies modulate the detection of simple auditory targets (one or two pure tones). Indeed, they report that auditory perception depends on the timing - i.e. phase - of the visual input. A separate EEG experiment suggests that visual information drives auditory processing, indicated by stimulus-related neural activity in visual regions leading that in auditory ones. The approach taken by the authors is highly interesting and timely, and could be a major influence in the corresponding research field. However, several issues with the paper, raised in my previous review, persist. These are described below.

1) The authors describe their findings as evidence for theta oscillations "organising information flow across visual and auditory sensory areas". I wonder whether the results reported can simply be explained by the fact that visual and auditory systems are connected with each other, i.e. visual input will also be transmitted to auditory regions, and vice versa. If the authors provide quasi-rhythmic input to the visual system (in the form of lip movements), then this information will also be relayed to the auditory system - potentially with a certain time lag (reaching the visual system first, given that the stimulus is visual). If the two systems are connected, then it is likely that input from one modality will influence the processing of simultaneous information in the other. It might therefore not be very surprising that the detection of auditory targets depends on the timing of simultaneous visual input: For instance, auditory detection might be worse when visual input is particularly informative. If the latter changes rhythmically, then its influence on the former will also be rhythmic.

Here, the reviewer suggests that the theta synchronisation observed between visual and auditory systems is a trivial side effect of visual entrainment by lip movements. In particular the reviewer suggests that due to the interconnectivity between auditory and visual processing regions, any signal present in one modality (i.e. visual) is passively relayed to the other modality (i.e. auditory), where it may affect performance. This is indeed an important point that needs consideration. Assuming that visual information passively propagates to the auditory system, any time lag between the visual and auditory cortex would be constant across time. This is because any signal present in the visual cortex, regardless of whether it is an ERP or a quasi-rhythmic stimulus, would show up in the auditory cortex after a fixed delay dictated by synaptic transduction delays. We directly tested this hypothesis by analysing theta phase lags between early and late time windows within trials. If passive signal transmission Detailed Response to Reviewers were responsible for the observed effects in the auditory system then we would predict the theta phase lags to be similar between early and late time windows. However, the results of this analysis refute this idea in showing that the phase lags differ between early and late time windows. These results show that the phase lags between visual and auditory cortex decrease over time, which suggests an active process that increases the synchronization between the two systems as across the trial.

We highlighted this point in the Results section (**P10-11** and **Figure 4C-D**), as well as in a paragraph of the Discussion (**P13-14**; **lines 319-343**), where we provide these two possible interpretations on the dynamic theta synchronisation between the visual and auditory areas.

The authors state that "[their] results show that the theta phase lag between auditory and visual cortices decreases across time, which speaks against a process that would just passively relay activity from visual to the auditory cortex". However, the first (but not the second) time window will be influenced by neural responses to stimulus onset. Steady-state responses to rhythmic stimuli

typically take some time until they follow the input rhythm in a more stable fashion. The fact that the phase lag between auditory and visual regions changes over time might simply reflect the neural response becoming more stable after an initial period influenced by stimulus onset, and does not necessarily provide evidence for an active mechanism preparing the auditory system for upcoming information. Indeed, the fact that the phase of lip movements modulates auditory detection even in a scenario in which visual and auditory information are unrelated, seems to speak against such a mechanism, and rather for a more automatic one.

We acknowledge the concern raised by the reviewer. However, we believe her/his argument could not account for the EEG results reported in the present study (see response to the first point of this reviewer). Firstly, we exclude any influence of early ERP components evoked in the early time window by analysing phase coupling from + 0.5 to + 2 seconds after the stimulus onset (see theta phase coupling analysis section in the Methods section (**P32-33; lines 870-897**). Secondly, it has been shown that the occipital cortex entrains rapidly to a visual flickering stimulation (10 Hz), with a strong phase locking of the even-related potential settling in within 300 ms after stimulus onset (see Figure below; taken from Hanslmayr et al., 2011 in *Brain Research Reviews*).

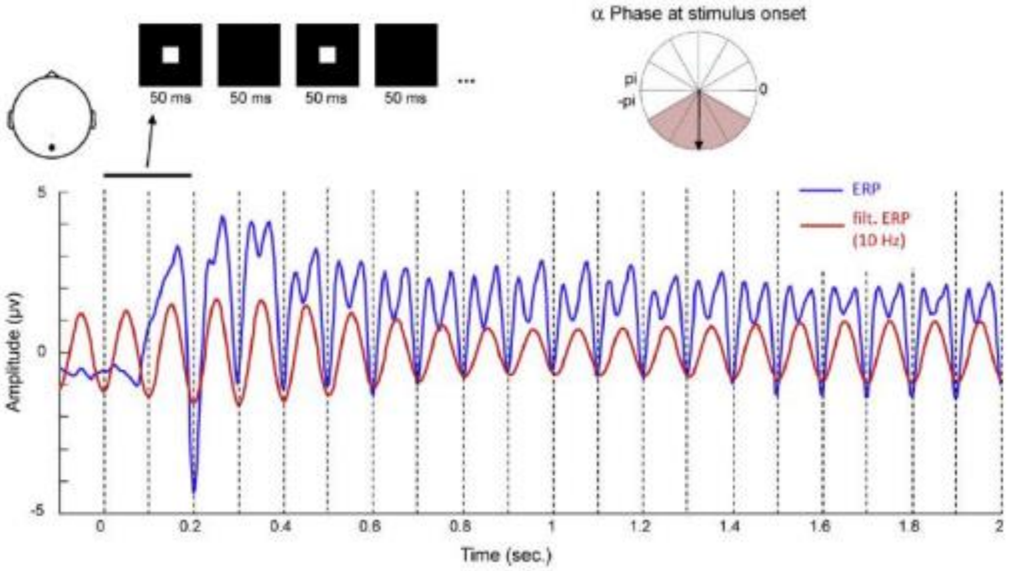

Fig. 5 – The ERP data (electrode Oz; averaged over 17 participants) is shown during a steady-state visual potential experiment. A stimulus (square) flickering at 10 Hz was presented. The blue line shows the unfiltered ERP, and the red line shows the bandpass-filtered ERP (8–12 Hz). Note, the constant negative phase at stimulus onset.

Therefore, we can assume that the visual source is already well entrained to lip movements at 500 ms, i.e. the start of our analysis time window. It follows that the phase offset reflects the degree of synchrony between the visual and auditory systems during visual entrainment in both the early and late windows. We therefore can safely interpret the decrease of phase lag as an estimate of the dynamic adjustment of synchrony between the two sensory systems over time, instead of a simple difference between noise (early) and entrainment (late window). Furthermore, the reviewer points out that the phase of lip movements modulates auditory detection of unrelated information, which suggests an automatic mechanism. The present study tested whether visual entrainment to lip

movements modulates auditory perception, which was assessed with tone detection. From our point of view, this does not contradict our interpretation in any way because speech perception depends, amongst other things, on basic auditory perception. Therefore, any process that modulates auditory perception arguably would also have knock-on effects on speech perception. However, we acknowledge that we did not test for effects of visual entrainment on speech perception per se and therefore cannot conclude that it does influence speech perception. This is an interesting question for future research, which we highlight in the Discussion section (see **P17; lines 421-433**).

In addition, it is well known that auditory perception is optimal when preceded by visual input within a certain time window (30-100 ms) - see for instance work by Thorne & Debener, Diederich & Colonius. It seems to me that the authors replicate this work, with the only difference being that their visual information fluctuates quasi-rhythmically - leading to benefits on auditory perception that are also quasi-rhythmically. Together, I believe that these issues should be given much more prominence in the paper.

We thank the reviewer for this reference that we have integrated in the manuscript and we comment in our discussion as follow (**see P14; lines 358-362**):

"Thorne et al. [42] associated cross-modal phase resetting with neural temporal predictions occurring when rhythmic visual input precedes auditory input within a stable time-window (~30 to 100ms). Our phase coupling results are in line with this finding [42], and suggest that the visual and auditory systems adjust over time to maintain this optimal delay in the cortex as well, potentially to allow for neural temporal predictions."

2) **I was previously confused by the structure of the Results section and the description of statistical analyses, and I still am. It seems to me that the analysis of the main hypothesis - that auditory detection is modulated by the timing (i.e. phase) of lip movements - is hidden in the text. Instead, the authors start by testing whether the distribution of the phase of lip movements during detected auditory targets is non-uniformly distributed across participants. This test does not reveal the magnitude (or indeed, the presence) of a phasic modulation, but instead whether all participants have the same "preferred" visual phase (leading to highest auditory accuracy). The latter could be true even in the absence of a significant phasic modulation. Conversely, even if preferred phases are uniformly distributed, there can still be a phasic effect, but with different "preferred" phases across participants.**

We thank the reviewer for this comment and we respond to this very first point here in a separate reply for clarity. Within-subject designs allowed testing for statistical effects on two levels, as there were repeated measures per condition for each subject. On the first level, one can test whether the phasic modulation is present in a given subject, while on the second level, one can test whether the phasic modulation is present across subjects. In the present study, we did the latter as a main analysis and the former as a secondary analysis, which we initially reported accordingly in the Result section. Nevertheless, we agree that a random phase distribution across subjects does not automatically implicate that there is no entrainment on the single subject level. Indeed, each subject can be significantly entrained to a different phase and in the case that we did not find entrainment across subjects, this possibility could not be fully excluded. However, we do not think that this argument applies in the opposite direction: in the case that results reveal no visual entrainment at subject level (i.e. noise), how would one expect phase locking across subjects? Our reply to the next part of the

reviewer's comment shows how we integrated her/his suggestions to solve this issue and to address visual phasic modulation estimate assuming or not assuming a different preferred phase across participants.

In my view, the results section needs to start with a statistical quantification of phasic modulation for each of the two tones separately, and in the one tone condition (followed by the comparison across tones). By adapting the analytical approach accordingly, this can be done with or without the assumption that all participants have the same preferred phase. In the Results section, the authors mention this assumption. In the last paragraph of the Discussion, they provide an argument against it: "one could assume that our results may reflect similar anatomical differences that influence how participants synchronize to visual lip activity and how information travels from visual to auditory cortex." Consequently, providing results for both might be the easiest solution. For example, in the authors' "control analysis" (which in my view should rather be a main analysis), they could leave out the alignment step (step two) to include the assumption of identical preferred phases across participants. Alternatively, the authors could fit a sine wave to the data (auditory detection as a function of visual phase) and extract its magnitude as an indicator of phasic modulation, with the phase of the sine wave fixed (assuming identical preferred phases) or variable (assuming different preferred phases) across participants.

We thank the reviewer for providing constructive comments on how to structure the Results section. We acknowledge that it is relevant to provide results on the phase modulation at both subject level (i.e. individual phase locking difference between hit and miss trials, assuming different phases across participants), and across participants (i.e. assuming that stimuli induce the same preferred phase across participants). In the new version of the manuscript, we made a significant reorganisation of the corresponding Result section to integrate the reviewer's suggestions, and to provide results from the two analytic approaches. We also state the motivation for using each of the approaches. We start with a statistical quantification of phasic modulation on the realigned individual phase for each of the two tones separately as well as between tones, in the two tones condition (Zoefel et al., 2019: https://doi.org/10.1016/j.neuroimage.2019.116175). This approach resolves the reviewer's suggestion regarding the individual phasic modulation estimate, although we did not directly calculate the individual vector lengths for the reason explained in our reply to the next comment (i.e. unbalanced number of hit/miss trials). This approach stands now as a main result with respect to the reviewer's suggestion. We also added a figure (Figure 2) to illustrate these new results in the new manuscript. Second, we report results from the statistical quantification of phasic modulation across participants for each of the two tones separately as well as between tones, in the two tones condition. This approach was initially the main approach reported in the previous manuscript and stand now as the second analytic approach in the new manuscript. The same two analyses are also applied on the single tone condition (individual phase locking and across participants), and reported in the supplementary information. In the previous version, we stated that the single tone condition served to control for tone predictability and was not initially designed to test for the phasic modulation. We also pointed out the fact that there were half of the trial numbers as compared to the two tones condition, and thus, less statistical power. Further, we improved the corresponding Methods section in the new manuscript where we describe the two analytic approaches. We hope that we have addressed the reviewer's concerns in a satisfactorily manner.

Please see **P5-8; lines 111-199** (Result section) and **P25-27; lines 677-740** (Methods section) in the revised manuscript, as well as revised Supplementary Information (**P1-3; lines 3-79**).

Finally, the reviewer suggests that the discussion regarding the behavioural difference between the two sub-groups of participants (sorted according to their mean phase), contradicts the assumption that all participants have the same preferred phase. We acknowledge this point and we made clear in the discussion that this interpretation was speculative and data-driven. We added a short note on it with respect to the reviewer's comment, as follows (**see P16; lines 409-411**):

"Nevertheless, this data-driven interpretation contrasts somehow with our initial assumption of a similar mean phase modulation induced in the brain by the same stimuli across participants, which will need to be unravelled in the future."

From the authors' description, it is currently still unclear to me whether vector length is computed separately for each participant (quantifying phasic modulation of auditory detection for each participant) or for the distribution of mean phases across participants (testing whether these are uniformly distributed). As mentioned above, this makes a big difference for the conclusion that can be drawn from the outcome.

We thank the reviewer for this comment and are happy to clarify. Vector length was computed for the distribution of mean phases across participants only, in the previous version of the manuscript. With respect to the reviewer's comment above, we now report results in which the phasic modulation between hit and miss trials is quantified individually after realigning the phase of subjects on their preferred phase. In this approach, we did not use the resultant vector length but the approach described by Zoefel et al. (2019) to quantify visual entrainment modulation between hits and misses across tone positions. The main reason is that the tone detection task was calibrated to reach an individual threshold (i.e. targeting ~75 per cent of correct responses in the tone detection task), with a significant difference of hit and miss trials within subjects. As the resultant vector length depends on the number of trials, the individual vector length of the miss trials results artificially greater than for the hit trials.

Indeed, we performed permutation tests on the individual vector length, similarly to those applied on the mean phases across participants described in the manuscript (not reported). To calculate the original effect size before performing the permutation tests, we applied two approaches to compensating the unbalanced number of hit/miss trials for each participant: (1) Randomly subsampling the number of hit trials with the available number of miss trials, or (2) Adding random hit trials to the miss trials to reach the same number of hit/miss trials before calculating the effect size in each condition. However, the consistency of the results was weak in both cases and we preferred to adopt the approach reported in the manuscript, which is more robust (i.e. realigning the phase and assess phasic modulation).

3) A minor point which I would ask the authors to consider: I wonder whether indeed the visual influence on auditory information processing builds up over time during speech processing. Wouldn't that mean that we struggle to understand each beginning of a sentence?

This is an interesting point and we thank the reviewer for this. If the visual filter builds up over time, this would be theoretically the case that the listeners struggle more to understand the early part of sentences until the auditory system synchronises and tunes to improve speech processing.

Nevertheless, this is not the case in most of speech perception conditions as (1) auditory signal often provides enough information to achieve comprehension without visual information. (2) Sentences are rarely processed in isolation in real life and previous semantic context allows generating predictions that facilitate comprehension about the following sentences. Therefore, it is difficult to estimate how visual filtering "ignition" delay accounts for a loss of understanding at the beginning of sentences. We added a short comment regarding to this point in the new version of the manuscript (**see P15; lines 426-433**):

"Further, if a visual filtering builds up over time as hypothesized here, one may argue that listeners would struggle more to understand the beginning of sentences until the auditory system synchronises and tunes to improve speech processing. Nevertheless, this is not the case in most of speech perception conditions as (1) auditory signal often provides enough information to achieve comprehension without visual information. (2) Sentences are rarely processed in isolation in real life, and previous semantic context allows generating predictions that facilitate next sentence comprehension. These are interesting questions for future research."

Comments from Reviewer 2

This paper describes two experiments (a behavioural and an EEG) investigating the effect of silent visual speech on auditory perception. In the behavioural study participants detected tones embedded in noise whilst looking at silent videos of speakers producing speech. Participants were better and faster at detecting tones when they occurred later in the course of the trials. Furthermore, the phase of the ongoing lip aperture signal at the onsets of successfully detected tones was more consistent later in the course of the trials. This suggested that the quasi-rhythmic lip movements in the silent speech videos may have entrained the visual and eventually the auditory system aligning its excitability with the visual signal. The EEG experiment confirmed this hypothesis demonstrating that the visual speech signal entrained brain activity predominantly in left auditory and visual areas. Phase coupling analysis revealed that the phase of the neural activity in visual areas consistently preceded the phase in auditory areas confirming that visual areas entrained by the silent speech signal modulated activity in auditory areas. These results provide further evidence supporting the hypothesis that visual speech signals improve speech perception via oscillatory mechanisms. Critique: The objective and main question of the study is timely and interesting. The results represent a relevant contribution to our understanding of the mechanisms of how visual speech signals improve speech perception. I could not identify any serious flaws with the study design and execution. Statistical methods were adequately chosen. The results are adequately discussed in light of a broader body of literature. Figures are well designed, informative and include individual data points. Accordingly, I only have minor comments, mainly aimed at improving clarity, which I list below.

I- Minor Comments

1. Figure 2:

a. Panel B y-axis tickmarks: 4 units are divided into 3 equal parts between 1.5 and 5.5, this way it is very hard to judge the values. I suggest spacing tickmarks 1 unit apart, so it is easier to judge the

values

We agree with the reviewer and we adapted the graphs accordingly in the new version of the manuscript.

b. Boxplots throughout the paper: it is not clear if the SEM is depicted by the shaded area around the mean or the box? I suspect the latter, but then what do the shaded grey areas around the mean values represent? Please clarify.

We thank the reviewer for asking to clarify. The upper and bottom sides of each box depict the mean ± standard deviation. The shaded grey area around the mean within the box represents the standard error of the mean. The interval between the upper and bottom error bars outside the box indicate the 5th and 95th percentiles. We clarified the boxplots in the legends of the corrected manuscript.

2. I thought that the control experiment described between lines 142 and 160 might be better placed in the supplement, however I do not have strong feelings about this, it is up to the Authors to decide.

We actually increased the relevance of this analysis in the new version of the manuscript, with respect to the comment #2 from the other reviewer. Please see **P5-8; lines 111-199** (Result section) and **P25-27; lines 677-740** (Methods section) in the revised manuscript, as well as revised Supplementary Information (**P1-3; lines 3-79**).

3. Line 184-185: It is not clear how this statement follows from the results presented immediately before. Please clarify.

This statement summarizes the fact that the tone position (first vs. second window) predicts detection performance (accuracy d' and reaction times). We reformulated this statement in the new manuscript: "Therefore, the tone position predicted performances in the TDT (d' and reaction times)."

4. Figure 3:

a. Panel A: please indicate the z coordinate of the slice depicted either in the figure or in the legend (I believe it is z = 0)

The reviewer is correct and we have now added the z coordinate of the illustrative slice in the figure caption.

b. I think it would be informative to indicate the maximal auditory and visual voxels with markers on panel A using the colour coding in panel B.

Agreed. We have added a pink and green dot to localise visually the maximal auditory and visual voxels respectively in the new manuscript.

5. Line 653-655: I did not fully understand why this step was performed the way it is described. Could you please explain in more detail?

We acknowledge this part needed to be unpacked. This step allows quantifying the effect size (phasic modulation) between hit and miss trials after realigning the phase on the preferred bin. Instead of taking directly the distance between the peaks at the most preferred phase (white dot in the illustrative figure below, taken from Zoefel et al., 2019), we take "areas" to measure the effect size taking into account a larger number of phases. Effect size: the maximal response was aligned to the centre bin and the remaining bins phase-wrapped. The average response of the two bins adjacent to the bin opposite to the centre bin (red dots) was subtracted from the average response of the two bins adjacent to the centre bin (green dots).

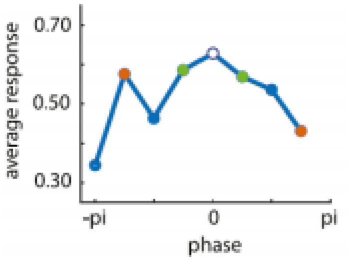

We have reformulated this part in the new manuscript with an appropriate reference describing the analytic approach from Zoefel et al. (2019), as well as a figure (Figure2). Please, **see P25; lines 677-690** in the Methods section.

6. Line 770: Please indicate which regions were actually included in the auditory and visual areas

We now indicate the auditory and visual regions in the new manuscript: "[…] in regions corresponding to the auditory (left Middle Temporal cortex) and visual areas (left Middle Occipital cortex), and defined using the automated anatomical labelling atlas (AAL, [67])."

7. Line 790: It is confusing which auditory and visual signals are you referring to here. I suspect they are the reconstructed source level time series activities measured at voxels exhibiting maximal MI with the lip aperture signal in auditory and visual areas respectively. Please define this here more precisely.

The reviewer is correct and we added her/his suggestion to clarify this part in the new manuscript version (**P32; lines 870-873**):

"First, auditory signal was projected orthogonally onto the visual signal (i.e. the reconstructed source level time-series activities measured at voxels exhibiting maximal MI with the lip movement signal in auditory and visual areas respectively) applying a Gram-Schmidt process (GSP; [69]) for single trials before computing phase information."

8. Line 806: The phase slope index calculation is introduced to control for potential circularity

issues. Could you please explain briefly here how the phase slope index helps controlling for these issues?

In the present manuscript, we assessed the directionality of information flow between visual and auditory systems through the sign of the theta phase difference over time. This approach is valid here because we have only two sources and supposedly a predominant unidirectional interaction (i.e. visual to auditory source direction). However, in non-ideal circumstances (e.g. bidirectional interaction), this estimate can be misleading because the phase difference is circular modulo 360° (or 2π). For instance, + 90° (π/2) and - 270° (-3π/2) phase offsets are the same in a particular frequency, and it becomes difficult to infer any directionality. To circumvent circularity issue without previous knowledge about the directionality, the phase slope index (PSI) computes the consistency of the direction of the change in the phase difference across the frequencies from a band centred on the frequency of interest. For each frequency, the PSI computes the change in the phase difference between the neighbouring frequencies, weighted with the coherence. When the phase difference changes consistently across frequencies (i.e. increases with frequency bin distance within the band), and there is substantial coherence, the PSI deviates from zero. The sign indicates which signal temporally leads the other one (for further details, see Bastos & Schoffelen, 2016 in *Front. Syst. Neurosci*).

We hope this explanation helped to clarify here why we applied this control approach. Accordingly, we added a relevant reference and further details to explain the principle of PSI in the corresponding part of the Methods section in the corrected manuscript (**see P32; lines 887-897**).

II- Typos:

1. Line 85-86: "… silent movies centred on the speakers' nose displayed with sound albeit non-informative". I think some words are missing here, please reformulate.

We agree with the reviewer and we reformulated this part as follows (**P4; lines 85-88**):

“To test the first hypothesis of visual entrainment affecting auditory processing, a random movie was displayed with the sound in each trial. Participants were asked to attend carefully to the silent movies centred on the speakers’ nose, albeit non-informative to perform the tone detection task.”

2. Line 88: "mouth apertures" instead of "mouth's apertures"
Corrected.

3. Line 96: "In each trial" instead of "For each trial"
Corrected.

4. Line 97: "for five seconds" instead of "during five seconds"
Corrected.

5. Line 107: "proportion" instead of "percentage"

Corrected.

6. Line 109: "random uniform distribution" instead of just "random distribution".

Corrected.

7. Line 110 and 112: please use either "tone onset" or "tone's onset" consistently Corrected. We went carefully through the whole manuscript to adjust when needed and keep it consistent with "tone onset".

8. Line 182: Please use either Fig. or Figure consistently when referring to figures in the text.

Corrected to Figure in the whole text.

9. Line 196: "… and tone detection improved both in the late window…" It is not clear to me what "both" is referring to here. Please clarify.

We agree with the reviewer and we clarified this as follows (see **P9; lines 226-228**):

"The effect of visual entrainment to theta lip activity on auditory perception was established by (1) the phasic modulation that predicted performance in the late window and, (2) the improvement of tone detection in the late window."

10. Line 203: "Sensitive" instead of "sensible"

Corrected.

11. Line 217: "… contrasting the difference of MI between the early and late time window" I may have misunderstood this but to me the sentence would make sense either as: "... contrasting the difference of MI between the early and late time window with zero" or just simply "…contrasting the MI between the early and late time window". The same issue appears in line 744 as well.

The reviewer is right and we corrected this part with "[…] contrasting the MI between the early and late time window" where necessary in the new manuscript.

12. Line 312: "with" instead of "within"

Corrected.

13. Line 398: a brief reference of where to find the relevant details would be helpful here. Something like: for details see Tone detection performances section.

We have added this reference accordingly in the new manuscript.

14. Line 505: "tone detection" instead of "tones detection"

Corrected.

15. Line 509: "consisted of" instead of "consisted in"

Corrected.

16. Line 567: "practice" instead of "practiced"
Corrected.

17. Line 585: "…to reflect the direction of the auditory system…" This sounds a bit odd, please reformulate.
Agreed. We reformulated this part with: […] to reflect the modulation of the auditory system by the entrained visual system to lip movements."

18. Line 638: "… to determine a resultant vector length…" I would perhaps write "…to determine the resultant vector length across participants..." Adding "across participants" would help understanding.
We agree with the reviewer, and we added "across participants" when needed in the new manuscript.

19. Line 664: "where" instead of "were"
Corrected.

20. Line 754: "realigned with respect to" instead of "realigned respect to"
Corrected.

III- Comments to the Custom Review Question(s) point D: The data required to replicate the results were not deposited to open data repositories at the time of the review but there is a statement that the data will be made available after publication of the manuscript.
That is correct. The data and associated scripts were deposited to open repositories that will be made accessible after publication (i.e. github.com and osf.io).

2nd Decision letter

Reference: CRNEUR-D-20-00016
Title: Auditory detection is modulated by theta phase of silent lip movements
Journal: Current Research in Neurobiology

Dear Dr. Biau,

Thank you for submitting your manuscript to Current Research in Neurobiology.

I have completed my evaluation of your manuscript. The reviewers recommend reconsideration of your manuscript following minor revision and modification. I invite you to resubmit your manuscript after addressing the comments below. Please resubmit your revised manuscript by Apr 30, 2021.

When revising your manuscript, please consider all issues mentioned in the reviewers' comments

carefully: please outline every change made in response to their comments and provide suitable rebuttals for any comments not addressed. Please note that your revised submission may need to be re-reviewed.

Current Research in Neurobiology values your contribution and I look forward to receiving your revised manuscript.

Kind regards,

Anna S Mitchell, Ph.D.
Editor in Chief
Current Research in Neurobiology

Comments from Editors and Reviewers:

Reviewer #1:

I'd like to thank the authors who have very thoroughly addressed my comments and clearly improved their manuscript.
There are however a few remaining points which I think need clarification: In particular, I struggled to understand the analysis yielding results shown in Figure 2 - I believe this is mostly due to missing details. I hope the following questions can clarify the issues:

- The rationale why the authors re-align data separately for hit and miss trials could be better explained.

- The phrase "[…] the […] distribution […] was re-aligned on the maximum phasic modulation […]" (Methods) confused me: Such a modulation can only be tested by contrasting performance across bins.

- Why would the authors expect a "statistical difference in entrainment between hit and miss trials" (Methods)? Given the hypothesis of a modulation of tone detection performance by visual phase, would we not expect *both* the probability of hits and misses to be modulated by visual phase? [This issue also concerns another analysis (p. 7 in version with tracked changes): "we tested the difference of resultant vector length between hit and miss trials across participants for the first and second tones". Why compare results between hit and miss trials - a phasic modulation of performance should affect them both?]

- If I understand correctly, the authors first quantify the phasic modulation for hit and miss trials separately (performance in bins neighbouring the aligned bin vs that in opposite bins), and then compare the two. But if both hits and misses are expected to be modulated by phase, what does the comparison show us? It might be helpful to list the predicted outcome from each of the analytical steps if the hypothesis were true.

- It might make results shown in Fig. 2 easier to follow if the authors used the probability of correctly detecting the tone (i.e. n_hits / (n_hits+n_misses)) or sensitivity (i.e. d-prime) as a single measure of

performance instead of using two separate ones (hits and misses). If the preferred phase (highest hit probability, or highest d-prime) is used for re-alignment, then a phasic modulation would predict performance in the bins next to the aligned bin to be better than that in the opposite bins. If the worst phase (lowest hit probability etc) is used, then this would predict the opposite result.

- According to the authors' response letter, the first 500 ms were excluded from analyses (ruling out more trivial explanations for the change in phase lag between visual and auditory regions), but page 10 in the manuscript still defines the early time window as 0-2.5s. Which is correct?

2nd Author Response Letter

Response to comments from Editors and Reviewers:

We thank the editor and the reviewer for her/his additional comments and suggestions, which we carefully considered. Please find our answers to each comment below, which indicate where corresponding adjustments have been made in the revised manuscript as well. We hope to have addressed every concern in a constructive manner and look forward to your decision.

Emmanuel Biau and Simon Hanslmayr, on behalf of all the authors.

Comments from Reviewer 1

I'd like to thank the authors who have very thoroughly addressed my comments and clearly improved their manuscript. There are however a few remaining points which I think need clarification: In particular, I struggled to understand the analysis yielding results shown in Figure 2 - I believe this is mostly due to missing details. I hope the following questions can clarify the issues:

1. The rationale why the authors re-align data separately for hit and miss trials could be better explained.

We agree with the reviewer and we acknowledge that this segment needed to be unpacked. We clarified the rationale behind the re-aligned data separately for hit, and miss trials in the corrected version of the manuscript as follows below. Please, see our reply to the comment #3 that addresses the related concern in more detailed explanations:

P.5; line 117: “Second, we compared the amplitude of the phasic modulation between correctly detected (hit trials) and missed tones (miss trials) to test how visual entrainment may account for auditory detection performances at the first and second tones. We hypothesized that visual entrainment shapes auditory perception by tuning auditory activity more consistently towards a more optimal state for when the tones’ onsets occur, and increasing their detection. In contrast, a more inconsistent tuning of the auditory system reflected by a weaker phasic modulation might lead to missing more tones. Therefore, we expected the phase modulation to predict behavioural outcomes, with a greater phase modulation in hit trials as compared to miss trials. If true, this would be the case only during the second tone window but not during the first tone window, i.e. when visual entrainment though lip movement perception has built up sufficiently.”

2. The phrase "[…] the […] distribution […] was re-aligned on the maximum phasic modulation […]" (Methods) confused me: Such a modulation can only be tested by contrasting performance across bins.

We agree that the second step in the analysis needs to be clarified. After obtaining the distribution of accuracy across trials along the visual phase, we determined the phase bin yielding the best performance as the "preferred phase" by means of automatic peak detection (i.e., visual phase bin with the highest accuracy). The bin position of the preferred phase was shifted to the centre bin (binpreferred phase = 20/2+1) and the remaining bins phasewrapped accordingly. This operation was computed for hit and miss trials separately. We clarified this step of the analysis in the corrected version of the manuscript:

P.25; line 713: "Second, after obtaining the distribution of accuracy across trials along the visual phase, the "preferred phase" bin yielding the best performance was determined by means of an automatic peak detection (i.e., visual phase bin with the highest accuracy). The preferred phase bin position was shifted to the centre bin (binpreferred phase = 20/2+1) and the remaining bins were phase-wrapped accordingly. This operation was computed for hit and miss trials separately."

3. Why would the authors expect a "statistical difference in entrainment between hit and miss trials" (Methods)? Given the hypothesis of a modulation of tone detection performance by visual phase, would we not expect *both* the probability of hits and misses to be modulated by visual phase? [This issue also concerns another analysis (p. 7 in version with tracked changes): "we tested the difference of resultant vector length between hit and miss trials across participants for the first and second tones". Why compare results between hit and miss trials - a phasic modulation of performance should affect them both?]

We thank the reviewer for giving us the opportunity to clarify this point. We hypothesized that visual entrainment shapes auditory perception by tuning activity of the auditory system more consistently towards an optimal state at the tones' onsets that improves their detection. Therefore, we expected the phase modulation to predict behavioural outcomes, with a greater phase modulation in hit trials as compared to miss trials. If true, this would be the case only during the second tone window, i.e. once visual entrainment through perception of lip movement has built up. Nevertheless, we agree that there could be a phase modulation in the miss trials as well, i.e. with a distinct preferred angle tuning auditory system to a less optimal state when tones' onsets occurred and leading to missing more tones as compared to hit trials. However, in miss trials participants may have missed auditory tones for additional reasons (e.g., attentional blink, fatigue etc.), which perturbed also visual entrainment stability and its influence on auditory system activity. Therefore, the resultant phase modulation in the miss trials embedded either less optimal phase and noise, or the same phase as for hits but with more variability across trials. In contrast, participants correctly detected tones in hit trials because they successfully attended to both visual and auditory stimulus streams until the onset of the tones, representing the case of maximum likelihood to entrain visually to lip movements (i.e., stronger phasic modulation tuning auditory system to a more optimal state at tones' onsets). Consequently, we expected a stronger visual entrainment in the hit trials as compared to the miss trials, leading to a greater phase modulation in the realigned phase data. With respect to the second analytic approach (P.7), we tested the same hypothesis by comparing the resultant vector length

instead (i.e., reflecting consistency toward a mean preferred phase), as we assumed that a similar mean phase emerged across participants. For the exact same reasons as for the realigning phase approach, we expected visual entrainment to be smaller in the miss trials, resulting from a less consistent and noisier modulation of auditory system activity at missed tones' onsets compared to detected tones' onsets. Indeed, the results from both measures support the hypothesis of a greater phase modulation towards a preferred phase and a longer resultant vector length for the hit trials in the late second tone window. This corroborates our interpretation of stronger visual entrainment predicting performances in the later second tone window. We have added a clarification on the motivation to compare results between hit and miss trials in the new version of the manuscript as follows:

P.5; line 117: "[…] Second, we compared the amplitude of the phasic modulation between correctly detected (hit trials) and missed tones (miss trials) to test how visual entrainment may account for auditory detection performances at the first and second tones. We hypothesized that visual entrainment shapes auditory perception by tuning auditory activity more consistently towards a more optimal state for when the tones' onsets occur, and increasing their detection. In contrast, a more inconsistent tuning of the auditory system reflected by a weaker phasic modulation might lead to missing more tones. Therefore, we expected the phase modulation to predict behavioural outcomes, with a greater phase modulation in hit trials as compared to miss trials. If true, this would be the case only during the second tone window but not during the first tone window, i.e. when visual entrainment though lip movement perception has built up sufficiently."

P.8; line 195: "As for the previous analytic approach, we compared hit and miss results to establish whether visual entrainment accounted for auditory detection performances differently in the first and second tone windows. If the visual system entrained more efficiently to lip movements leading to improve tones' detection in the hit trials, we expect a longer resultant vector length at the detected tones' onsets as compared to missed tones. In line with the results from the first analytic approach, we expect to find a greater vector length for the hit trials as compared to miss trials only in the second tone window when visual entrainment has built up through perception of lip movement."

4. If I understand correctly, the authors first quantify the phasic modulation for hit and miss trials separately (performance in bins neighbouring the aligned bin vs that in opposite bins), and then compare the two. But if both hits and misses are expected to be modulated by phase, what does the comparison show us? It might be helpful to list the predicted outcome from each of the analytical steps if the hypothesis were true.

The reviewer is correct regarding the analytic steps to compute the phasic modulation and compare it between hit and miss trials. In line with our reply to the previous comment #3, the comparison between hits and misses established whether successfully detected tones were associated with a greater phase modulation (i.e., more efficient visual entrainment to lip activity) as compared to the tones that were missed. Second, we expected this to be true later in the trials when visual entrainment was more stable in the second tone window, but not in the earlier first tone window. Please see our previous reply for the related adjustments implemented in the new version of the manuscript (comment #3).

5. It might make results shown in Fig. 2 easier to follow if the authors used the probability of

correctly detecting the tone (i.e. n_hits / (n_hits+n_misses)) or sensitivity (i.e. d-prime) as a single measure of performance instead of using two separate ones (hits and misses). If the preferred phase (highest hit probability, or highest d-prime) is used for re-alignment, then a phasic modulation would predict performance in the bins next to the aligned bin to be better than that in the opposite bins. If the worst phase (lowest hit probability etc) is used, then this would predict the opposite result.

We thank the reviewer for her/his relevant comment. In line with our hypothesis that visual phase modulation predicts tone detection, we think that the analyses comparing the probability of both the detected and missed tones along the visual phase remains relevant in the manuscript. Nevertheless, we acknowledge that it is relevant to test in a first step the existence of a significant phase modulation when participants effectively detected the tones in each condition. Following the reviewer's suggestion, we computed the probability of correctly detecting the tone, i.e., p(hits) = n_hits / (n_hits + n_misses) as a single measure of performance. As in our previous analysis, phasic modulation was estimated by subtracting the average response of the two bins adjacent to the bin opposite to the preferred phase (highest p(Hits)) from the average response of the two bins adjacent to the preferred phase. The existence of a significant phase modulation at the first and second tone in each condition was assessed statistically by comparing the mean phasic modulations against zero by applying one-sampled T-tests (one-tailed; p-value adjusted for multiple comparisons with a Bonferroni correction). Results revealed a strong tendency of a significant phase modulation at the second tone of the two tones condition ($t(1,23) = 2.356$; $p = 0.055$; Bonferroni-corrected), but not at any other tone (first tone of the single tone condition: $t(1,23) = -1.035$; $p = 1$; second tone of the single tone condition: $t(1,23) = -1.444$; $p = 1$; first tone of the two tones condition: $t(1,23) = -0.046$; $p = 1$; Bonferroni-corrected). Although the corrected p-value is slightly above $\alpha = 0.05$, this result supports the existence of a phase modulation driving the correct detection of the second tones but not the earlier first tones in the condition of interest. We now report this result in the new version of the manuscript. Further, we modified the Figure 2 to report p(hits) = n_hits / (n_hits + n_misses) along the visual phase, according to the reviewer's suggestion.

P.5; line 127: "As a first step, we tested the existence of a significant phase modulation when participants effectively detected the tones in each condition (hit trials only). We computed the probability of correctly detecting the tone p(hits) = hits / (hits + misses) as a single measure of performance. Phasic modulation was computing by subtracting the average response of the two bins adjacent to the bin opposite to the preferred phase, i.e. with the highest p(hits), from the average response of the two bins adjacent to the preferred phase (Figure 2). The existence of a significant phase modulation at the first and second tone in each condition was assessed statistically by comparing the mean phasic modulations against zero by applying independent one-sampled T-tests (one-tailed; p-value adjusted for multiple comparisons with a Bonferroni correction). Results revealed a strong tendency for the mean distance to be significantly greater than zero at the second tone ($t(1,23) = 2.356$; $p_{adjusted} = 0.055$; Cohen's $d = 0.481$), but not at earlier first tone ($t(1,23) = -0.046$; $p_{adjusted} = 1$; Cohen's $d = -0.001$). Although the p-value corrected for multiple comparisons was slightly above $\alpha = 0.05$, this result suggests the existence of a phase modulation driving the correct detection of the second tones but not the earlier first tones in our condition of interest."

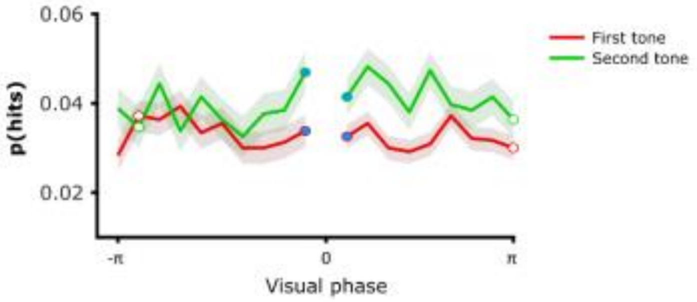

Figure 2. Phasic modulation along the realigned phase in the two tones condition. Probability of correctly detected tones p(hits) = hits / (hits + misses) along the visual phase realigned on the preferred bin (0° bin, not plotted) in the two tones condition. The red line depicts p(hits) at the first tone and the green line depicts p(hits) at the second tone (mean ± standard deviation). The phase modulation was estimated by subtracting the average response of the two bins adjacent to the bin opposite to the preferred phase (white dots) from the average response of the two bins adjacent to the preferred phase (blue dots).

P.2; line 63 (Supplementary Information): "First, we computed the probability of correctly detecting the tone p(Hits) = hits / (hits + misses) as a single measure of performance, for tones sorted as first and seconds in the single tone condition (Figure S2). We assessed the existence of a phase modulation at detected sorted first and second tones (i.e. hit trials) by testing the mean distance against zero for hit trials at each tone (first and second tones) with one-sample T-tests as previously for the two tones condition (mean phase modulation against zero; onetailed; p-values adjusted with a Bonferroni correction for multiple comparisons). Results revealed that the mean distance was not significantly greater than zero at the first tones (t(1,23) = -1.035; padjusted = 1; Cohen's d = -0.211) and the second tones (t(1,23) = -1.444; padjusted = 1; ; Cohen's d = -0.295). This result suggested the absence of phase modulation driving the correct detection of the first and second tones in the single tone condition (Figure S2)."

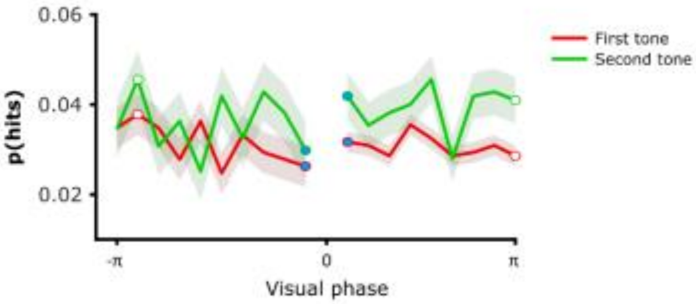

Figure S2. Phasic modulation along the realigned phase in the single tone condition. Probability of correctly detected tones p(Hits) = hits / (hits + misses) along the visual phase realigned on the preferred bin (0° bin, not plotted) in the single tone condition. As previously, each single tone was sorted as a first or a second tone according to its onset. The red line depicts p(hits) at the first tone and the green line depicts p(hits) at the second tone (mean ± standard deviation). The phase modulation was estimated by subtracting the average response of the two bins adjacent to the bin opposite to the preferred phase (white dots) from the average

response of the two bins adjacent to the preferred phase (blue dots).

6. According to the authors' response letter, the first 500 ms were excluded from analyses (ruling out more trivial explanations for the change in phase lag between visual and auditory regions), but page 10 in the manuscript still defines the early time window as 0- 2.5s. Which is correct?

For the sake of reading flow, we referred to the early window MIearly as 0-2.5s in the main body to correspond with the first tone window reported in the tone detection task (without detailing the sub-windows in which we computed the mutual information [0.5-2.5s] or phase coupling [0.5-2s] analyses in the main text). With respect to the reviewer's suggestion, we added further details in the Material and Methods section of the corrected manuscript:

P.32; line 862: "For each single trial, MI was first computed separately in the time-windows equivalent to the first (0.5 to 2.5 seconds after trial onset; MIearly) and second tone timewindows of the TDT (2.5 to 4.5 seconds after trial onset; MIlate) at the 2020 virtual electrodes […] The first and last 500 ms at the edges of the epoch were not included into MI analyses to avoid the trial onset and offset responses."

3rd Decision letter

Reference: CRNEUR-D-20-00016
Title: Auditory detection is modulated by theta phase of silent lip movements
Journal: Current Research in Neurobiology

Dear Dr. Biau,

Thank you for submitting your manuscript to Current Research in Neurobiology.

I have completed my evaluation of your manuscript. The reviewers recommend reconsideration of your manuscript following minor revision and modification. I invite you to resubmit your manuscript after addressing the comments below. Please resubmit your revised manuscript by Jun 05, 2021.

When revising your manuscript, please consider all issues mentioned in the reviewers' comments carefully: please outline every change made in response to their comments and provide suitable rebuttals for any comments not addressed. Please note that your revised submission may need to be re-reviewed.

Current Research in Neurobiology values your contribution and I look forward to receiving your revised manuscript.

Kind regards,

Anna S Mitchell, Ph.D.

Editor in Chief

Current Research in Neurobiology

Comments from Editors and Reviewers:

We've finished the reviews of your manuscript. We all concur that it is an excellent piece of research. We'd like to provisionally accept your manuscript. Congratulations!
Please can you adjust the one sentence in the text based on the one additional point made by the reviewer. In addition, the editorial team suggested your media press statement could be improved so it is more understandable to our general audience readership.
I look forward to receiving your final adjusted version as soon as you have it ready.

Reviewer #1:

I thank the authors for their continuous efforts to improve this manuscript. They clearly report an important dataset. However I do feel the need to insist on one final point - but would be happy to be corrected. I struggle with the following statement: "We hypothesized that visual entrainment shapes auditory perception by tuning activity of the auditory system more consistently towards an optimal state at the tones' onsets that improves their detection."

By definition, if auditory perception entrained to the silent lip movements, then this would not only lead to an optimal state for auditory detection (at the "up" of the oscillation), but also to a non-optimal state (at the "down"). The authors argue that stronger entrainment would lead to more hits - but I would rather think that it leads to a more pronounced alternation between "good" and "poor" phases for auditory perception, and therefore to a stronger modulation of perception in general (i.e. for both hit and miss trials), and not only for hits.

Let's assume we are in a state of strong visual entrainment. As the onset of target tones is unpredictable, the target might fall at a "good" phase of the entrained oscillation and be likely to be detected, or at a "poor" phases and less likely to be detected. Strong visual entrainment would therefore lead to a more pronounced alternation between states in which hits and misses are more likely, respectively, whereas weaker entrainment leads to perception that is more stable in time.

I appreciate that the authors now also show a phasic modulation of auditory detection in general (i.e. combining hits and misses), which further adds to the quality of this manuscript - but I do think the point above is worth clarifying.

3rd Author Response Letter

Response to comments from Editors and Reviewers:

We thank the editor and the reviewer for the last comments, which we addressed. Please find our answer to the reviewer’s comment and the corresponding adjustments made in the revised manuscript below. We took this opportunity to add two additional one-sample t-tests to establish that the resultant vector length r of the distance between the observed ϕA-V and the zero ϕA-V was

greater than zero in both the early and late windows, and confirm that visual activity led auditory activity during the trials **(P12; line 323)**. We also corrected the mean angle values reported in Figure 3C-D, and updated the corresponding plots accordingly. We hope to have addressed it satisfyingly and look forward to your decision.

Emmanuel Biau and Simon Hanslmayr, on behalf of all the authors.

Reviewer 1:

I thank the authors for their continuous efforts to improve this manuscript. They clearly report an important dataset. However, I do feel the need to insist on one final point - but would be happy to be corrected. I struggle with the following statement: "We hypothesized that visual entrainment shapes auditory perception by tuning activity of the auditory system more consistently towards an optimal state at the tones' onsets that improves their detection." By definition, if auditory perception entrained to the silent lip movements, then this would not only lead to an optimal state for auditory detection (at the "up" of the oscillation), but also to a non-optimal state (at the "down"). The authors argue that stronger entrainment would lead to more hits - but I would rather think that it leads to a more pronounced alternation between "good" and "poor" phases for auditory perception, and therefore to a stronger modulation of perception in general (i.e. for both hit and miss trials), and not only for hits. Let's assume we are in a state of strong visual entrainment. As the onset of target tones is unpredictable, the target might fall at a "good" phase of the entrained oscillation and be likely to be detected, or at a "poor" phases and less likely to be detected. Strong visual entrainment would therefore lead to a more pronounced alternation between states in which hits and misses are more likely, respectively, whereas weaker entrainment leads to perception that is more stable in time. I appreciate that the authors now also show a phasic modulation of auditory detection in general (i.e. combining hits and misses), which further adds to the quality of this manuscript - but I do think the point above is worth clarifying.

We thank the reviewer for this relevant comment, which gives us the opportunity to clarify this statement. We indeed expected the phase modulation to predict behavioural outcomes, with a greater phase modulation in hit trials as compared to miss trials. This would be the case during the second tone window when visual entrainment has built up through lip movement perception. We acknowledge that in principle there could exist a similar phase modulation in the miss trials as well, with a distinct preferred phase angle reflecting a less optimal state at onsets and leading to missing more tones. Consequently, this should increase the alternation between optimal and non-optimal states in which hits and misses respectively are more likely (as suggested by the reviewer). However, it is important to keep in mind that a miss likely has many reasons, non-optimal phase being just one. For instance, a participant might not detect the auditory tone because of lapses of attention, or fatigue, or a distracting thought. All of these would affect visual entrainment strength and its influence on auditory system activity. Therefore, the resultant phase modulation in the miss trials likely embedded Detailed Response to Reviewers not only non-optimal phases but also a considerable amount of trials where the tone was presented at the optimal phase but missed for other reasons. In contrast, participants correctly detected tones in hit trials because they successfully attended to both visual and auditory stimulus streams until the onset of the tones, representing the case of stronger phasic modulation in our procedure. In consequence, we expected a stronger visual entrainment in the hit trials as compared to the miss trials. Finally, we targeted a mean correct response rate of 70-80% in the tone detection task (by determining the individual threshold in the preceding calibration

task). Our paradigm was therefore not designed to analyse the phasic modulation in the miss trials because we focused primarily on the influence of visual entrainment on auditory perception in the successful trials. The absence of phasic modulation in the miss trials could thus also result from weaker statistical power as compared to the hit condition. Nevertheless, we acknowledge that the statement in the previous version of the manuscript needed to be unpacked and we added some clarifications as follows:

P.5; line 120: "We hypothesized that visual entrainment shapes auditory perception by tuning auditory activity more consistently towards a more optimal state for when the tones' onsets occur, and increasing their detection. In contrast, we expected inconsistent tuning of the auditory system at the onsets of the missed tones reflected by a weaker phasic modulation. One might argue that a similar phase modulation in the miss trials exists as for hit trials, where the auditory system is entrained to a non-optimal phase. However, it is important to note that participants may have not detected auditory tones for a number of reasons like attentional lapses, fatigue, or distracting thoughts. Consequently, the resultant phase modulation in the miss trials embedded either the same preferred phase as for hits but with more variability across trials, or a distinct preferred phase angle reflecting a less optimal state when tones' onsets occurred and leading to missing more tones. Therefore, we expected the phase modulation to predict behavioural outcomes, with a greater phase modulation in hit trials as compared to miss trials."

Accept Letter

Dear Dr. Biau,

I am pleased to tell you that your manuscript has been accepted for publication in Current Research in Neurobiology. Congratulations!

Your accepted manuscript will now be transferred to our production department. We will create a proof which you will be asked to check, and you will also be asked to complete a number of online forms required for publication. If we need additional information from you during the production process, we will contact you directly.

We appreciate and value your contribution to Current Research in Neurobiology. We regularly invite authors of recently published manuscript to participate in the peer review process. If you were not already part of the journal's reviewer pool, you have now been added to it. We look forward to your continued participation in our journal, and we hope you will consider us again for future submissions.

Thank you again for trusting us with your science,

Kerry Walker, DPhil
Associate Editor
Current Research in Neurobiology

-------- End of Review Comments --------
